# Supplementary material for: Impact of decoding strategies on GPU energy usage in large language model text generation
Source: Sci Rep. 2025 Dec 11;16:2168. doi: 10.1038/s41598-025-31896-0 (PMC12808663; doi:10.1038/s41598-025-31896-0)
Supplement: Supplementary file 1 — Supplementary Information. [file 41598_2025_31896_MOESM1_ESM.pdf]

Supplementary Information

Table S5. Optimal Hyperparameters for the Decoding Strategies used in the experiments with Qwen2.5-7B-Instruct. The Greedy decoding method does not require any hyperparameters, so we represented it with a dash (-) in the table.

Dataset	Deterministic Methods						Stochastic Methods					
	Greedy	Beam	DBS	DoLa	CS	AD	τ	Top-p	Top-k	Eps	Typical	Min-p
De→En	-	5	(10,2)	"low"	k=25, α=0.2	5	0.1	0.3	5	0.004	0.5	0.9
En→De	-	10	(10,2)	"low"	k=5, α=0.2	2	0.1	0.3	5	0.004	0.5	1.0
GSM8K	-	2	(4,2)	"low"	k=5, α=0.4	2	0.1	0.95	5	0.004	0.95	0.05
HumanEval	-	2	(4,2)	"low"	k=5, α=0.2	5	0.5	0.3	100	0.002	0.5	0.5
WikiText	-	2	(4,2)	"low"	k=5, α=0.2	5	0.3	0.7	50	0.0006	0.5	0.3

Table S6. Optimal Hyperparameters for the Decoding Strategies used in the experiments with Llama3.1-8B-Instruct. The Greedy decoding method does not require any hyperparameters, so we represented it with a dash (-) in the table.

Dataset	Deterministic Methods						Stochastic Methods					
	Greedy	Beam	DBS	DoLa	CS	AD	τ	Top-p	Top-k	Eps	Typical	Min-p
De→En	-	5	(4,2)	"high"	k=5, α=0.2	2	0.1	0.3	5	0.004	0.5	0.7
En→De	-	10	(4,2)	"low"	k=5, α=0.2	2	0.1	0.3	5	0.004	0.5	0.9
GSM8K	-	5	(4,2)	"low"	k=5, α=0.2	2	0.7	0.3	5	0.0009	0.8	0.7
HumanEval	-	10	(10,2)	"low"	k=5, α=0.2	10	0.1	0.5	5	0.002	0.5	0.9
WikiText	-	2	(4,2)	"low"	k=5, α=0.4	2	0.7	0.5	50	0.002	0.9	0.02

Table S7. Optimal Hyperparameters for the Decoding Strategies used in the experiments with Qwen2.5-14B-Instruct. The Greedy decoding method does not require any hyperparameters, so we represented it with a dash (-) in the table.

Dataset	Deterministic Methods						Stochastic Methods					
	Greedy	Beam	DBS	DoLa	CS	AD	τ	Top-p	Top-k	Eps	Typical	Min-p
De→En	-	2	(10,5)	"low"	k=5, α=0.2	5	0.1	0.3	5	0.004	0.5	1.0
En→De	-	10	(10,2)	"low"	k=25, α=0.2	2	0.1	0.3	5	0.004	0.5	0.9
GSM8K	-	2	(5,5)	"low"	k=25, α=0.2	2	0.5	0.3	5	0.004	0.8	0.9
HumanEval	-	10	(10,2)	"low"	k=25, α=0.2	10	0.5	0.7	5	0.004	0.8	0.5
WikiText	-	10	(4,2)	"high"	k=5, α=0.2	10	0.5	0.7	25	0.004	0.5	0.7

Table S8. Text Generation Quality and Average Energy Consumption across Decoding Methods in their best Hyperparameter Setting using Llama3.1-8B-Instruct with batch size=1. The associated hyperparameters are listed in Table S6. The table uses color coding to highlight key metrics: blue indicates high generative quality, red signifies high energy consumption, and green represents a high efficiency ratio. ↑ indicates higher is better, ↓ indicates lower is better

Task	Metric	Deterministic Methods						Stochastic Methods					
		**Greedy**	**Beam**	**DBS**	**DoLa**	**CS**	**AD**	τ	**Top-p**	**Top-k**	**Eps**	**Typical**	**Min-p**
De→En	$BLEU\uparrow$	47.07	47.77	47.56	32.96	47.31	47.02	46.91	47.50	31.94	33.49	38.31	47.18
	$Wh\downarrow$	9.26	10.89	10.48	18.44	11.00	8.34	9.32	9.38	11.02	10.50	11.05	9.18
	$ER\uparrow$	5.08	4.38	4.53	1.78	4.30	5.63	5.03	5.06	2.89	3.18	3.46	5.13
En→De	$BLEU\uparrow$	33.03	35.35	34.67	19.36	32.99	32.90	33.08	33.49	21.14	19.18	24.91	33.42
	$Wh\downarrow$	13.40	16.95	14.89	27.10	15.41	12.19	13.42	13.42	14.71	14.69	14.18	13.18
	$ER\uparrow$	2.46	2.08	2.32	0.71	2.14	2.69	2.46	2.49	1.43	1.30	1.75	2.53
GSM8K	$Exact-M\uparrow$	0.77	0.78	0.76	0.66	0.78	0.75	0.75	0.80	0.61	0.62	0.66	0.76
	$Wh\downarrow$	8.16	15.61	13.85	12.42	13.58	6.54	8.15	8.36	8.39	9.57	8.73	7.86
	$ER\uparrow$	0.094	0.049	0.054	0.053	0.057	0.114	0.092	0.095	0.072	0.064	0.075	0.096
HumanEval	$pass@1\uparrow$	0.71	0.78	0.77	0.67	0.72	0.72	0.69	0.70	0.48	0.51	0.61	0.71
	$Wh\downarrow$	6.10	16.89	14.99	8.48	7.33	5.27	6.13	4.85	7.67	8.13	6.98	6.03
	$ER\uparrow$	0.116	0.046	0.051	0.079	0.098	0.136	0.112	0.144	0.062	0.062	0.087	0.117
WikiText	$MAUVE\uparrow$	76.87	88.5	76.12	95.54	95.72	82.6	95.88	95.5	97.37	95.73	96.95	95.2
	$Wh\downarrow$	76.83	82.53	89.05	112.23	94.30	54.54	77.28	77.74	77.47	77.68	78.04	78.41
	$ER\uparrow$	1.00	1.07	0.85	0.85	1.01	1.51	1.24	1.22	1.25	1.23	1.24	1.21

Table S9. Text Generation Quality and Average Energy Consumption across Decoding Methods in their best Hyperparameter Setting using Qwen2.5-14B-Instruct with batch size=1. The associated hyperparameters are listed in Table S7. The table uses color coding to highlight key metrics: blue indicates high generative quality, red signifies high energy consumption, and green represents a high efficiency ratio. ↑ indicates higher is better, ↓ indicates lower is better

Task	Metric	Deterministic Methods						Stochastic Methods					
		**Greedy**	**Beam**	**DBS**	**DoLa**	**CS**	**AD**	τ	**Top-p**	**Top-k**	**Eps**	**Typical**	**Min-p**
De→En	$BLEU\uparrow$	37.60	43.79	43.44	28.06	37.04	39.18	37.42	37.54	30.24	30.72	36.70	38.02
	$Wh\downarrow$	19.41	18.44	24.15	35.21	22.80	16.44	19.47	19.55	19.98	20.06	18.85	19.11
	$ER\uparrow$	1.93	2.37	1.79	0.79	1.62	2.38	1.92	1.92	1.51	1.53	1.94	1.98
En→De	$BLEU\uparrow$	27.33	30.50	30.32	23.78	27.20	27.24	27.32	26.67	20.43	19.23	22.89	27.17
	$Wh\downarrow$	23.80	30.67	31.10	35.51	35.04	22.47	24.24	23.86	24.34	24.81	23.88	23.93
	$ER\uparrow$	1.14	0.99	0.97	0.66	0.77	1.21	1.12	1.11	0.83	0.77	0.95	1.13
GSM8K	$Exact-M\uparrow$	0.84	0.84	0.84	0.83	0.87	0.76	0.85	0.86	0.79	0.84	0.85	0.86
	$Wh\downarrow$	37.07	42.53	69.54	54.07	192.8	25.14	38.91	37.58	36.49	36.62	37.70	37.32
	$ER\uparrow$	0.022	0.019	0.012	0.015	0.004	0.030	0.021	0.022	0.021	0.022	0.022	0.023
HumanEval	$pass@1\uparrow$	0.69	0.87	0.86	0.66	0.69	0.68	0.73	0.78	0.58	0.65	0.72	0.71
	$Wh\downarrow$	6.04	13.05	19.58	10.67	11.05	5.16	7.71	7.05	7.57	8.51	7.31	6.69
	$ER\uparrow$	0.114	0.066	0.043	0.061	0.062	0.131	0.094	0.110	0.076	0.076	0.098	0.106
WikiText	$MAUVE\uparrow$	74.93	87.99	86.38	86.10	76.30	76.93	80.26	84.52	87.73	86.10	86.12	87.39
	$Wh\downarrow$	135.65	186.74	155.48	199.43	160.20	106.86	135.13	136.30	134.44	137.01	135.17	134.06
	$ER\uparrow$	0.55	0.47	0.55	0.43	0.47	0.71	0.59	0.62	0.65	0.62	0.63	0.65

Table S10. Relative Standard Deviation (RSD) and Sharpe Ratio (SR) for each decoding strategy in Llama3.1-8B-Instruct. RSD is calculated for text generation quality and average energy consumption across different hyperparameter values. SR takes into account the quality and energy consumption of all hyperparameter runs within a decoding method. ↑ indicates higher is better

Task	Metric	Deterministic Methods						Stochastic Methods					
		**Beam**	**DBS**	**DoLa**	**CS(k=5)**	**CS(k=25)**	**AD**	τ	**Top-p**	**Top-k**	**Eps**	**Typical**	**Min-p**
De→En	*BL (RSD%)*	0.34	0.51	2.29	12.80	34.92	1.67	34.97	13.71	4.18	4.07	7.31	12.53
	Wh (RSD%)	9.21	7.73	2.11	15.13	53.21	2.06	32.68	8.07	1.85	3.03	2.11	5.94
	SR ↑	$1.2e^{-2}$	$1.4e^{-2}$	$2.2e^{-2}$	$6.3e^{-3}$	$8.3e^{-4}$	$7.4e^{-2}$	$2.5e^{-3}$	$1.3e^{-2}$	$3.8e^{-2}$	$2.5e^{-2}$	$4e^{-2}$	$1.9e^{-2}$
En→De	*BL (RSD%)*	0.69	1.53	0.15	7.06	22.00	4.71	45.50	18.17	8.08	4.87	14.24	16.80
	Wh (RSD%)	7.41	6.33	0.15	1.75	13.38	4.36	36.82	4.29	1.08	1.42	3.90	5.14
	SR ↑	$8.5e^{-3}$	$8.9e^{-3}$	$7.8e^{-2}$	$2.9e^{-2}$	$2.6e^{-3}$	$1.6e^{-2}$	e^{-3}	$1.2e^{-2}$	$3e^{-2}$	$2.2e^{-2}$	$9.9e^{-3}$	$1.1e^{-2}$
GSM8K	*EM (RSD%)*	1.62	5.28	3.12	3.53	28.33	14.25	35.09	8.26	4.80	6.29	4.69	7.02
	Wh (RSD%)	33.02	24.71	2.66	15.00	40.85	8.68	32.70	1.37	6.02	0.37	1.84	2.92
	SR ↑	$3.8e^{-5}$	$4.1e^{-5}$	$5.3e^{-4}$	$8.6e^{-5}$	$8e^{-6}$	$3.3e^{-4}$	$5.2e^{-5}$	$1.5e^{-3}$	$2.7e^{-4}$	$3e^{-3}$	e^{-3}	$7.8e^{-4}$
HumanEval	*ACC (RSD%)*	2.72	4.69	0.00	6.16	41.79	11.55	32.57	9.30	11.04	4.66	7.15	12.49
	Wh (RSD%)	50.41	43.17	0.63	10.38	80.34	18.34	94.56	17.19	9.05	8.95	11.12	15.66
	SR ↑	$4.1e^{-5}$	$5.4e^{-5}$	$2.8e^{-3}$	$2.7e^{-4}$	$7e^{-6}$	$2.1e^{-4}$	$1.2e^{-5}$	$1.4e^{-4}$	$1.6e^{-4}$	$1.6e^{-4}$	$1.8e^{-4}$	$1.5e^{-4}$
WikiText	*MAU (RSD%)*	6.92	7.56	0.60	3.49	35.98	2.04	44.56	1.12	2.32	2.09	2.37	8.59
	Wh (RSD%)	11.36	9.55	0.18	1.92	6.72	5.98	0.24	0.20	0.08	0.22	0.27	0.38
	SR ↑	$2.3e^{-3}$	$2e^{-3}$	$15e^{-2}$	$2e^{-2}$	$2e^{-3}$	$7.8e^{-3}$	$9.8e^{-2}$	$10e^{-2}$	$37e^{-2}$	$12e^{-2}$	$9.4e^{-2}$	$7.2e^{-2}$

Table S11. Relative Standard Deviation (RSD) and Sharpe Ratio (SR) for each decoding strategy in Qwen2.5-14B-Instruct. RSD is calculated for text generation quality and average energy consumption across different hyperparameter values. SR takes into account the quality and energy consumption of all hyperparameter runs within a decoding method. ↑ indicates higher is better

Task	Metric	Deterministic Methods						Stochastic Methods					
		**Beam**	**DBS**	**DoLa**	**CS(k=5)**	**CS(k=25)**	**AD**	τ	**Top-p**	**Top-k**	**Eps**	**Typical**	**Min-p**
De→En	*BL (RSD%)*	0.49	0.16	30.84	16.83	28.36	1.41	14.65	10.19	5.18	2.92	10.81	7.33
	Wh (RSD%)	10.30	7.61	26.00	18.41	34.15	1.84	4.40	4.81	2.02	2.33	5.99	2.22
	SR ↑	$5.4e^{-3}$	$6.8e^{-3}$	$4.6e^{-4}$	$1.7e^{-3}$	$5.4e^{-4}$	$3.3e^{-2}$	e^{-2}	$9.6e^{-3}$	$1.7e^{-2}$	$1.6e^{-2}$	$7.2e^{-3}$	$2.2e^{-2}$
En→De	*BL (RSD%)*	1.48	2.62	20.19	14.05	45.42	4.69	24.81	12.85	7.60	3.68	8.44	10.49
	Wh (RSD%)	8.19	6.69	4.07	3.30	27.44	3.68	1.74	0.58	0.52	0.60	0.41	0.51
	SR ↑	$3.5e^{-3}$	$4e^{-3}$	$3.5e^{-3}$	$6.8e^{-3}$	$4.2e^{-4}$	$8.8e^{-3}$	$1.3e^{-2}$	$4.2e^{-2}$	$3.5e^{-2}$	$3.2e^{-2}$	$4.8e^{-2}$	$4.9e^{-2}$
GSM8K	*EM (RSD%)*	2.28	1.00	0.60	1.47	54.54	26.74	3.85	2.60	1.05	6.21	2.81	2.65
	Wh (RSD%)	35.11	23.10	0.61	0.88	1.75	9.36	0.62	1.15	0.16	0.50	0.99	1.09
	SR ↑	$9e^{-6}$	$1.2e^{-5}$	$6.4e^{-4}$	$3.6e^{-4}$	$4.7e^{-5}$	$7.4e^{-5}$	$8e^{-4}$	$4.7e^{-4}$	$1.1e^{-3}$	$7.5e^{-4}$	$5.3e^{-4}$	$5.3e^{-4}$
HumanEval	*ACC (RSD%)*	2.43	3.56	12.82	9.81	44.21	4.73	25.00	8.11	4.37	9.31	10.18	8.23
	Wh (RSD%)	22.65	25.40	45.22	5.44	77.28	13.97	34.15	8.75	4.97	9.38	4.42	12.05
	SR ↑	e^{-4}	$6.5e^{-5}$	$1.8e^{-5}$	$4.1e^{-4}$	$7e^{-6}$	$2.7e^{-4}$	$5.9e^{-5}$	$3.2e^{-4}$	$3.9e^{-4}$	$1.9e^{-4}$	$5.1e^{-4}$	$2.2e^{-4}$
WikiText	*MAU (RSD%)*	5.15	5.40	1.41	21.53	65.05	2.24	4.44	5.04	3.84	0.89	5.39	4.61
	Wh (RSD%)	10.98	8.71	0.26	0.68	1.90	2.03	0.40	0.45	0.67	0.48	0.42	0.88
	SR ↑	$1.3e^{-3}$	$1.5e^{-3}$	$5.5e^{-2}$	$1.4e^{-2}$	$2.3e^{-3}$	$1.2e^{-2}$	$4e^{-2}$	$4.5e^{-2}$	$3.2e^{-2}$	$4.1e^{-2}$	$4.2e^{-2}$	$1.9e^{-2}$

Table S12. Average Energy Consumption (watt-hour per query), Average Latency (seconds per query), Average GPU Power Draw (watts) and Average GPU Utilization (%) in Llama-3.1-8B-Instruct model in the best Hyperparameter for each method

Task	Metric	Deterministic Methods						Stochastic Methods					
		**Greedy**	**Beam**	**DBS**	**DoLa**	**CS**	**AD**	τ	**Top-p**	**Top-k**	**Eps**	**Typical**	**Min-p**
De→En	*Wh/query*	0.0185	0.0217	0.0209	0.0368	0.022	0.0166	0.0186	0.0187	0.022	0.021	0.0221	0.0183
	sec/query	0.630	0.768	0.776	1.09	0.835	0.584	0.638	0.644	0.753	0.719	0.760	0.632
	util (%)	91.0	91.0	89.0	92.8	86.8	92.2	91.4	91.4	95.6	95.4	91.2	95.2
	watt	105.73	102.09	97.2	120.83	94.82	102.87	105.04	104.89	105.33	105.07	104.6	104.45
En→De	*Wh/query*	0.0268	0.0339	0.0297	0.0542	0.0308	0.0243	0.0268	0.0268	0.0294	0.0293	0.0283	0.0263
	sec/query	0.911	1.17	1.10	1.61	1.18	0.854	0.917	0.921	1.00	1.00	0.978	0.904
	util (%)	91.0	91.4	89.0	92.8	87.0	92.4	91.2	91.6	95.2	95.8	95.0	95.4
	watt	105.9	103.63	96.88	120.66	93.66	102.83	105.33	104.89	105.78	105.25	104.36	105.0
GSM8K	*Wh/query*	0.0816	0.1561	0.1384	0.1242	0.1358	0.0654	0.0815	0.0836	0.0839	0.0957	0.0873	0.0786
	sec/query	2.63	4.68	4.44	3.61	4.47	2.39	2.71	2.74	2.81	3.19	2.945	2.63
	util (%)	96.0	95.0	93.0	97.0	91.0	92.4	96.2	96.0	96.0	96.8	96.6	97.0
	watt	111.44	119.9	112.27	123.83	109.35	98.41	108.16	109.63	107.23	107.86	106.81	107.64
HumanEval	*Wh/query*	0.0613	0.1689	0.1499	0.0848	0.0733	0.0527	0.0613	0.0484	0.0767	0.0813	0.0698	0.0603
	sec/query	2.06	5.79	5.02	2.60	2.78	1.93	2.11	1.67	2.66	2.79	2.44	2.08
	util (%)	93.8	86.0	91.6	96.0	87.0	91.4	93.2	93.6	94.8	93.0	94.8	93.2
	watt	106.27	104.97	107.48	117.44	94.79	98.32	104.51	104.34	103.78	104.7	102.72	104.5
WikiText	*Wh/query*	0.1920	0.2063	0.2226	0.2805	0.2357	0.1363	0.1932	0.1943	0.1936	0.1942	0.1951	0.1960
	sec/query	6.48	7.11	8.02	8.25	8.88	4.70	6.45	6.55	6.58	6.52	6.58	6.53
	util (%)	96.6	97.0	94.0	97.0	88.6	94.8	96.8	96.4	96.6	96.6	96.2	96.4
	watt	106.66	104.42	99.89	122.38	95.57	104.4	107.78	106.77	105.98	107.09	106.64	108.04

Table S13. Average Energy Consumption (watt-hour per query), Average Latency (seconds per query), Average GPU Power Draw (watts) and Average GPU Utilization (%) in Qwen2.5-14B-Instruct model in the best Hyperparameter for each method

Task	Metric	Deterministic Methods						Stochastic Methods					
		**Greedy**	**Beam**	**DBS**	**DoLa**	**CS**	**AD**	τ	**Top-p**	**Top-k**	**Eps**	**Typical**	**Min-p**
De→En	*Wh/query*	0.0388	0.0368	0.0482	0.0704	0.0456	0.0328	0.0389	0.0391	0.0399	0.0401	0.0377	0.0382
	sec/query	1.12	1.19	1.58	1.87	1.58	0.99	1.13	1.15	1.18	1.18	1.12	1.14
	util (%)	97.0	93.0	90.0	98.0	88.0	94.8	96.4	96.6	97.4	96.4	97.4	97.6
	watt	124.53	110.86	109.74	134.93	103.56	119.58	123.2	122.44	121.02	122.25	120.24	120.48
En→De	*Wh/query*	0.0476	0.0613	0.0622	0.0710	0.0700	0.0449	0.0484	0.0477	0.0486	0.0496	0.0477	0.0478
	sec/query	1.40	1.96	1.99	1.88	2.29	1.34	1.42	1.42	1.45	1.46	1.42	1.42
	util (%)	97.4	94.0	92.0	98.0	91.0	94.8	96.0	97.0	97.2	96.8	97.6	97.4
	watt	122.0	112.66	112.18	135.39	109.74	120.21	122.7	120.37	120.29	122.37	120.58	120.82
GSM8K	*Wh/query*	0.3707	0.4253	0.6954	0.5407	1.928	0.2514	0.3890	0.3757	0.3649	0.3661	0.377	0.3732
	sec/query	10.65	13.07	20.28	14.52	50.26	8.31	11.19	10.85	10.81	10.84	11.18	10.80
	util (%)	97.2	97.0	94.4	98.0	97.0	95.0	97.2	97.6	97.4	98.0	98.0	97.6
	watt	125.25	117.07	123.4	134.02	138.11	108.85	125.16	124.66	121.47	121.63	121.41	124.42
HumanEval	*Wh/query*	0.0604	0.1305	0.1957	0.1067	0.1105	0.0516	0.0771	0.0705	0.0757	0.0851	0.0731	0.0669
	sec/query	1.80	4.06	6.01	2.83	3.48	1.66	2.29	2.16	2.31	2.55	2.19	2.04
	util (%)	96.0	91.8	94.0	97.0	91.8	94.0	96.2	96.0	96.4	96.0	96.4	96.4
	watt	120.69	115.64	117.12	135.37	114.3	112.02	120.83	117.38	117.81	120.05	119.91	117.7
WikiText	*Wh/query*	0.3391	0.4668	0.3887	0.4985	0.4004	0.2671	0.3378	0.3407	0.3361	0.3425	0.3379	0.3351
	sec/query	9.90	14.13	12.47	13.43	13.64	8.36	9.91	10.03	10.09	10.08	10.26	10.12
	util (%)	97.6	98.4	96.0	98.0	89.8	96.6	97.6	98.0	98.0	98.0	97.2	97.6
	watt	123.3	118.95	112.17	133.6	105.66	115.01	122.7	122.24	119.88	122.29	118.5	119.17

Table S14. Per-Model Friedman Test Statistics for Quality and Energy Consumption values across 5 tasks ($df = 11$)

Model	Quality		Energy	
	X^2	p	X^2	p
Qwen-2.5-7B-Inst	28.37	0.00284	43.15	$1.02e^{-05}$
Llama-3.1-8B-Inst	21.27	0.0307	43.96	$7.38e^{-06}$
Qwen-2.5-14B-Inst	23.35	0.0158	41.80	$1.75e^{-05}$

Table S15. Per-Task Friedman Test Statistics for Quality and Energy Consumption values across 3 models ($df = 11$)

Task	Quality		Energy	
	X^2	p	X^2	p
De→En	30.90	0.00114	25.00	0.00912
En→De	29.41	0.00196	31.07	0.00107
GSM8K	19.99	0.0455	26.74	0.00502
HumanEval	26.97	0.00464	29.74	0.00174
WikiText	12.36	0.337	27.67	0.00364

Table S16. Statistical significance (p-values) of Kendall's Tau correlations between Latency and Energy rankings

Model	De→En	En→De	GSM8K	HumanEval	WikiText
Qwen-2.5-7B-Inst	$1.63e^{-05}$	$3.22e^{-07}$	$3.22e^{-07}$	$1.63e^{-05}$	$1.8e^{-03}$
Llama-3.1-8B-Inst	$4.99e^{-04}$	$3.22e^{-07}$	$3.22e^{-07}$	$1.63e^{-05}$	$1.07e^{-04}$
Qwen-2.5-14B-Inst	$3.18e^{-03}$	$2.4e^{-04}$	$5.32e^{-06}$	$5.01e^{-08}$	$1.38e^{-02}$
